# A prospective study of methamphetamine use as a predictor of high school non-attendance in Cape Town, South Africa

**DOI:** 10.1186/1747-597X-5-25

**Published:** 2010-10-21

**Authors:** Andreas Plüddemann, Alan J Flisher, Rebecca McKetin, Charles D Parry, Carl J Lombard

**Affiliations:** 1 Alcohol & Drug Abuse Research Unit, Medical Research Council, PO Box 19070, Tygerberg 7505, South Africa; 2 Division of Child and Adolescent Psychiatry, University of Cape Town, J-Block Groote Schuur Hospital, Observatory 7925, South Africa; 3 National Drug and Alcohol Research Centre, University of New South Wales, Sydney NSW 2052, Australia; 4 Alcohol & Drug Abuse Research Unit, Medical Research Council, PO Box 19070, Tygerberg 7505, South Africa; 5 Department of Psychiatry, University of Stellenbosch, South Africa; 6 Biostatistics Unit, Medical Research Council, PO box 19070, Tygerberg 7505, South Africa

## Abstract

**Background:**

This prospective study investigated the association between life-long methamphetamine and other drug use and high school non-attendance, in a sample of high school students in Cape Town, South Africa.

**Methods:**

A random sample of 1535 high school students completed a baseline questionnaire in 2006, and were asked to complete a follow-up questionnaire 12 months later. The questionnaire included questions on substance use, including tobacco, alcohol, methamphetamine and cannabis use, demographic factors, and questions relating to school attendance and performance.

**Results:**

Forty-three percent of the students surveyed at baseline did not complete a follow-up questionnaire after 12 months. Compared with students who were not using selected substances, an adjusted logistic regression model showed that life-time methamphetamine use in addition to other substances was significantly associated with non-attendance (OR = 2.58, 95% CI: 1.24 - 5.36) when other non-substance use factors (repeating a year at school and being older than the norm for current grade) were taken into account.

**Conclusions:**

Early identification of students with methamphetamine and other substance use problems, and a supportive rather than punitive school policy, may be valuable in improving high school completion and student retention rates.

## Background

Over the past six years South Africa, particularly the city of Cape Town, has experienced a sharp increase in the use of methamphetamine[1]. This was initially noted informally by anecdotal reports in the media and then more formally by increases in admissions for methamphetamine related substance use disorders to specialist substance abuse treatment centres in Cape Town, and later confirmed by high school surveys[2-5]. By 2006 over 40% of individuals admitted to various substance abuse treatment facilities in Cape Town reported methamphetamine as their primary substance of abuse. Within two years, methamphetamine had become the most common primary substance of abuse for those admitted for substance abuse treatment in the city, surpassing the previous dominance of alcohol. Admissions were concentrated among adolescents, over 60% of whom reported using methamphetamine as a primary substance of abuse[2]. High school surveys confirmed high levels of methamphetamine use among adolescents, with a life-time prevalence estimate of 12% in a survey of 4605 students, and 9% in a survey of 1561 students in 2006[3,4]. Methamphetamine is almost exclusively smoked in Cape Town, particularly among adolescents. While very little data exists, police sources have indicated that the drug is mostly manufactured in the country currently on a relatively large scale. Most of these methamphetamine labs have been identified by police in the province of Gauteng, which includes Johannesburg and Pretoria. The trade of the drug appears to be mostly controlled by highly organized criminal gangs who have their 'home base' and largest membership in Cape Town. These gangs also have a long history of drug trafficking and dealing.

Evidence is increasingly implicating methamphetamine as one of the most harmful illicit drugs. It can affect mental health, causing depression, anxiety and psychoses, as well as have prominent behavioural effects, including aggression and impulsivity[6]. Studies of methamphetamine users have also indicated cognitive impairment, including memory loss and concentration problems, and irreversible neuronal damage[7]. These potential side effects are perhaps of particular relevance to school-going adolescents, who are required to engage in cognitive tasks and conform to a certain convention of behaviour while attending school. Moreover, the negative impact of substance use on school performance has a carry-over effect into adulthood, impeding opportunities for tertiary education and being associated with lower income, unemployment and lower life satisfaction[8].

The potential impact of methamphetamine use on high school non-attendance is therefore an important issue for investigation. School attrition rates are a particular challenge in many developing countries, with the South African government estimating a number of years ago that up to 60% of children enrolled in primary schools, drop out before completing high school[9]. A longitudinal survey conducted in Cape Town high schools confirmed high dropout rates, finding a dropout rate of 55% from grade 8 to the final follow-up survey in grade 12[10]. This study found that school dropout was significantly predicted by absenteeism, poverty, and past month cigarette use, but not by past month alcohol use and life-time illicit drug (mostly cannabis) use. The study adjusted for a range of confounders, including gender, age and repeating a grade at school. At the time that this study was conducted (1997-2001), there was no indication or evidence of methamphetamine use in Cape Town. Another previous cross-sectional study of high school students in Cape Town by Flisher et al. had also shown absenteeism to be related to the use of cigarettes, alcohol and cannabis in the past month[11]. This study suggested that interventions aimed at improving school attendance should comprise a substance abuse prevention component.

For the purposes of our study "non-attendance" is a collective term for students who are either absent from school (absenteeism) for any reason or have dropped out of school (meaning they no longer attend school).

The aim of the present study was to investigate whether the emergence of methamphetamine use would play a significant role in predicting high school non-attendance, further investigating the findings in Flisher et al.'s study which indicated that illicit drug use did not predict high school dropout, while past month cigarette use did predict dropout[10]. A number of studies, identified in a systematic review, have implicated substance use in predicting high school dropout[12]. In terms of illicit drugs, these previous studies have mostly reported on associations between dropout or absenteeism and cannabis use, with some referring to 'other illicit drugs', 'hard drugs' or 'injecting drug use'. To our knowledge, none have investigated methamphetamine use specifically in relation to high school non-attendance. The current study examined whether methamphetamine use among high-school students in school predicted non-attendance at school 12 months later, and whether this effect was beyond that observed with cannabis or tobacco use.

## Methods

### Design and sampling strategy

The school population was all high schools (N = 54) in the South Educational District, one of four education management districts in the city of Cape Town. This district was believed to be the most affected by methamphetamine use at the time the study was designed, based on treatment demand data[2]. The district encompasses some of the poorest suburbs of Cape Town and is the district most affected by criminal gangs. However, analysis from another random survey of all schools in Cape Town later showed relatively small differences in life-time prevalence of methamphetamine use across school districts[3]. Fifteen schools were randomly selected from this population, such that the probability of selection was directly proportional to the number of students in the school. One class of approximately 35 students was randomly selected from each of grades 8 (majority aged 13-14 years), 9 (majority aged 14-15 years) and 10 (majority aged 15-17 years). The initial data were collected in July and August 2006 (Time 1), and a follow-up survey with the same students was attempted approximately one year later during July and August 2007 (Time 2). Most schools were surveyed within a two week period of being exactly one year later, with only one school being surveyed one year and three weeks later.

### Procedures

Questionnaires were administered by trained staff in a standardized way in a classroom setting without the presence of school staff. Students were seated in such a way as to preserve confidentiality. Personal Digital Assistants (PDAs) were used to administer the questionnaires and students were able to choose from one of three major local languages (English, Afrikaans and isiXhosa). Brief instructions on how to use the PDAs were provided as well as a few 'practice questions'. Students were provided with a sealable envelope containing a unique number. Students were asked to enter the number in the requested field on the PDA, and to write their name, the name of their school, their grade and the date on the front of the envelope. They were then asked to seal the envelope, and sign across the sealed flap, thereby ensuring their data remained anonymous. These sealed envelopes were collected and returned to the students during the follow-up survey. They were asked to open the envelopes and transfer the number onto their new questionnaire on the PDA. In this way, data were linked while preserving anonymity.

Each student provided informed assent. Parents were informed of the study by letter and given the opportunity to withdraw their child from the study. Of all students approached to participate in the initial survey, only 50 refused or were withdrawn from the study. Ethical clearance for the study was obtained from the University of Cape Town's Faculty of Health Sciences Research Ethics Committee.

### Measures

This study formed part of a broader investigation into methamphetamine use and various health issues. The complete set of measures used in this broader investigation are described elsewhere[4]. The main outcome measure for the present study was non-attendance at school, as indicated by the student not being present for the one-year follow-up interview. This measure of non-attendance reflects both school drop-out and absenteeism. As our survey was anonymous and we were hence unable to link the reason for absenteeism or drop-out to individual students, we decided to combine these students as one group. While we were not able to establish reliable information for all students we were able to determine that approximately 50% of those classified as 'non-attenders' had left their school. A previous study of high school drop out in Cape Town had also found no discernable difference between students who were absent and students who had dropped out, and had also combined these students for analysis purposes [10]. For the purposes of the present study, we focused on a number of demographic and substance use variables, and variables related to the potential for school dropout, including failing a year at school and being older than the normal age for the grade they were currently in. Students' age and gender were recorded. Socio-economic status (SES) was assessed by asking students to describe their 'living circumstances' against the following five categories: 'We don't have enough money for food', 'We have enough money for food, but not clothes', 'We have enough money for food and clothes, but are short of other things', 'We have the most important things, but few luxuries', and 'We have money for luxury goods and extra things'. Substance use measures covered tobacco, alcohol, cannabis, methaqualone, cocaine, heroin, ecstasy and methamphetamine. For each of these substances students were asked whether they had ever tried them, and whether they had used them in the past 12 months, past 30 days, and past seven days. The prevalence of methaqualone, cocaine, heroin and ecstasy use was very low (life-time use equalled 3% or less) and thus these substances were not considered for analysis. Students were also asked whether they had ever repeated a year at school due to failing examinations. For the purposes of the present study, we also judged whether students were the appropriate normal age for their grade at the first survey. Students whose age was at least one year older than the normal age for their grade were categorized as 'age greater than normal for grade', thereby creating a binary variable for being in an age appropriate grade. Above normal age for grade 8 was considered 15 years or older, grade 9 16 years or older and grade 10 17 years or older.

### Data analysis

Data were analysed using SPSS 16.0 and STATA 10. For the calculation of the substance use prevalence and other proportions' confidence intervals we took the study design (clustering at school level) into account in STATA's survey analysis settings. A logistic regression model, adjusted for the study design only, was performed to establish the association between non-attendance and methamphetamine use and various other variables. The outcome variable in these regressions was non-attendance (versus 'in school'). In an attempt to establish whether methamphetamine specifically contributed to non-attendance in a combined adjusted model, four categories of substance use were created for contrasting purposes based on data collected at Time 1: (1) students who did not report smoking cigarettes currently (in the past 7 days), and had never tried cannabis or methamphetamine, (2) students who reported smoking cigarettes currently but had never tried cannabis or methamphetamine, (3) students who reported smoking cigarettes currently and had tried cannabis at least once, and (4) students who reported smoking cigarettes currently and had tried both cannabis and methamphetamine. This categorization was used to try to factor in the use of multiple substances into our analysis. To avoid confounding, students who had tried cocaine, heroin, ecstasy or methaqualone together with any other drugs in the categories above were excluded from this analysis. The covariates included in this model were 'repeating a year at school' and 'age greater than norm for grade'. These were identified as confounders because they were significantly related to both the outcome and main predictor variables. The regression models were developed using a backward stepwise selection procedure. SES and gender were considered as confounding variables but were not found to be related to our main predictor and outcome variables used in the logistic regression model. Age was not included as a confounder as it correlated with 'age greater than norm for grade'. Therefore only one of these two variables could be used and it was decided that the latter variable would be more important to include. A measure of mental health was also considered as a covariate but it did not significantly improve the model fit (p < 0.05) and was therefore excluded.

## Results

A total of 1561 students completed the initial questionnaire in 2006. At the 12-month follow-up survey, 26 students completed questionnaires but did not enter their survey identification number correctly in the first survey, making it impossible to match their data from the follow-up survey. These students were excluded from the analysis, leaving a sample of 1535 students. Of these 874 (56.9%) completed the follow-up survey and the remaining 661 (43.1%) students were absent for the follow-up survey or had left the school and were thus considered to be non-attenders.

The final sample comprised slightly more females (53%) than males. The majority identified themselves as 'Coloured'(77%), followed by Black African (17%) and White (3%). [Note: The terms "white", "black", and "Coloured", became entrenched in the apartheid era. They refer to demographic markers and do not signify inherent characteristics. They refer to people of European, African and mixed (African, European and/or Asian) ancestry, respectively. These markers were chosen for their historical significance. Their continued use in South Africa is important for monitoring improvements in health and socio-economic disparities, identifying vulnerable sections of the population, and planning effective prevention and intervention programs.] Forty-three percent and 38% reported Afrikaans (a local derivative of Dutch and certain Eastern languages) and English respectively as their home language. Sixteen per cent reported isiXhosa (a local African language) as their home language. Based on the SES question, 19% of the students came from lower income households (i.e. they chose one of the first two options of the SES question), 54% came from middle income households (i.e. they chose one of the third or fourth options of the SES question), and 27% chose the highest category on the SES question. The mean age of the students was 14.9 years (SD = 1.34), ranging from 12 to 19 years. Almost 30% had repeated at least one year at school and 23% were older than the normal age for their grade (Table 1).

**Table 1:** Descriptive data for entire sample (N = 1535)

Variable	n*	% (95% confidence interval)
Gender	1527	
Male		46.9 (43.5 - 50.3)
Female		53.1 (49.7 - 56.5)
Age category	1505	
12-14 years		42.1 (34.4 - 50.3)
15 years		26.8 (22.8 - 31.3)
16 years		17.1 (13.6 - 21.4)
17 years or older		13.9 (8.9 - 21.0)
Methamphetamine use	1535	
Never		91.2 (88.1 - 93.6)
Life-time use (not past 12 months)		4.0 (2.9 - 5.7)
Past 12 months use		4.8 (3.3 - 6.8)
Cannabis use	1535	
Never		75.2 (70.9 - 79.1)
Life-time use (not past 12 months)		12.0 (9.8 - 14.6)
Past 12 months use		12.8 (10.4 - 15.7)
Current smoking (past 7 days)	1535	25.4 (20.6 - 30.2)
Current alcohol use (past 7 days)	1535	7.0 (4.9 - 9.0)
Ever repeated a year at school	1534	29.1 (23.3 - 35.8)
Dropout/absent status	1535	
Dropped out/absent		43.1 (37.8 - 48.5)
In school		56.9 (51.5 - 62.2)
Age > than norm for grade	1498	22.8 (13.5 - 32.0)

* values are less than 1535 owing to missing values

Confidence intervals adjusted for design of the study

At Time 1, 8.8% of the students had tried methamphetamine at least once in their life, with a slightly higher proportion (though not significant) for male students (9.8%) than female students (7.9%), and 4.8% had used in the past year (Table 1). A similar division was noted for the 25% of students who had used cannabis at least once, with 12% of student having used in the past year. A quarter of the students reported smoking cigarettes in the past seven days and 7% reported drinking alcohol during the seven days prior to the survey. Of the students who completed a follow-up questionnaire, 21 (2.4%) reported having tried methamphetamine at least once who did not report methamphetamine use at Time 1.

Table 2 shows the differences in prevalence of methamphetamine use for students that were in school versus those that were not present at Time 2 for a number of substance use variables and selected other potential risk factors for non-attendance. The table also shows unadjusted odds ratios, which showed that life-time methamphetamine use, life-time cannabis use, current smoking, repeating a year at school, and being older than the norm for current grade were all significantly associated with non-attendance at Time 2. An adjusted logistic regression model (described in the methods section) showed that students who were current smokers, students who were current smokers and had tried cannabis, and students who were current smokers and had tried both cannabis and methamphetamine were significantly more likely, namely two to two and a half times, to be classified as non-attenders than students who were not current smokers and had not tried cannabis or methamphetamine (Table 3). In this adjusted model being older than the normal age for current grade remained significantly associated with non-attendance, while repeating a year at school was no longer significant (Table 3). Further logistic regression analyses did however not show significant differences in the odds ratios between the various groups of substance users in Table 3.

**Table 2:** Prevalence rates stratified by school attendance status and odds ratios for substance abuse and selected covariates (N = 1535)

	Prevalence		Odds ratio (95% CI) p-value **Simple models, adjusted for design only**^**1**^
		
	Not in school % (95% CI)	In-school % (95% CI)	
Life-time meth. use (Reference category (Ref)): Never used meth)	12.4 (8.5 - 17.7)	6.1 (4.1 - 9.0)	2.19 (1.39 - 3.46) p < 0.01
Life-time cannabis use (Reference category (Ref)): Never used cannabis)	30.7 (23.3 - 39.3)	20.3 (17.6 - 23.2)	1.75 (1.22 - 2.49) p < 0.01
Current alcohol use (Ref: Not using alcohol currently)	7.7 (4.9 - 12.0)	6.4 (4.6 - 8.8)	1.22 (0.7 - 2.12) p > 0.05
Current smoking (Ref. Not smoking currently)	34.2 (26.0 - 43.4)	18.8 (14.9 - 23.4)	2.25 (1.45 - 3.48) p <0.01
Ever repeated a year at school (Ref. Never repeated a year)	37.7 (30.7 - 45.3)	22.7 (16.7 - 30.0)	2.07 (1.42 - 3.02) p < 0.01
Age > than norm for grade (Ref. Age normal)	32.2 (22.3 - 44.1)	15.7 (9.2 - 25.6)	2.55 (1.85 - 3.52) p < 0.01

^1 ^Logistic regression adjusted for survey design (p-values were from Wald t-tests, df = 14)

adjusted for selected variables and study design (N = 1265)

**Table 3:** Logistic regression model for school attendance status by different groups of substance users

Dropout/absent	n	Odds ratio	95% CI
(Reference category = Never used meth, never used cannabis and not current smoker)	974		
Current smoking, but never used meth or cannabis	150	2.16**	1.44 - 3.25
Current smoking + life-time cannabis use, but never used meth	125	1.94*	1.09 - 3.47
Current smoking + life-time cannabis use + life-time meth use	47	2.58*	1.24 - 5.36
Age > than norm for grade (Ref. Age normal)	1265	2.16**	1.60 - 2.93
Ever repeated a year at school (Ref. Never repeated a year)	1265	1.32	0.98 - 1.79

* Significant at p < 0.05 level

** Significant at p < 0.01 level

p-values were from Wald t-tests, df = 14

Note: Some cases excluded from model owing to missing values

## Discussion

The high rate of students who were not present at school 12 months after they were originally surveyed, can be compared with findings of a previous study conducted in Cape Town, showing similar rates of high school dropout[10]. This finding alone is of concern, particularly in a country where poverty is endemic and educational achievement could play a significant role in addressing economic disparities[12]. In addition to poverty, organized criminal gangs are also highly prevalent in the communities surveyed in the present study, with drug dealing forming a significant part of their criminal activity. Many adolescents join these gangs in order to survive and those who are no longer in school are particularly vulnerable[13].

The methamphetamine use prevalence found in our study indicated substantial use of this drug among high school students, although the life-time prevalence of 9% was slightly lower than the prevalence of 12% found in a study a year earlier in Cape Town covering a larger geographic area[3]. The past 12 months prevalence of 4.8% was similar to the 4.2% (past year amphetamine use for 12-17 year-olds) found in a national survey of schools in Australia in 2005, a country with one of the highest rates of methamphetamine use in the world[14,15].

Methamphetamine use in combination with other drugs doubled the odds of not attending school. Students who did not complete the survey at Time 2 were twice as likely to have reported life-time methamphetamine use at Time 1. This association remained significant in a regression model where multiple substance use was considered and with the inclusion of two confounding variables, namely repeating a year at school and being older than the expected normal age for current grade. These confounding variables have been found to be related to high school dropout in other studies[16]. A review by Townsend et al. similarly concluded a largely consistent relationship between dropping out of high school and substance use, although the studies in this review did not investigate methamphetamine specifically and studies differed in their findings of which substances were related to dropout[12]. Subsequent studies have continued to confirm this relationship, with a recent study finding a robust relationship between early cannabis use and educational attainment, including high school dropout[17].

Our study followed an 'additive approach' to substance use, rather than considering different substances as confounders of each other, as a number of previous studies have done. For example, a U.S. study by McCaffrey et al. found that the positive association between cannabis use and high school dropout became insignificant when measures of cigarette smoking were included in their analysis[18]. A study of high school dropout in Australia found that while current smoking was also a significant predictor of high school dropout, it did not diminish the association between weekly cannabis use and dropout for those students who left school in year 10 or year 11[19]. Flisher et al. (2010) found that current smoking had a stronger association with high school dropout than life-time illicit drug use, and Ellickson et al. (1998) found that in all ethnic groups except Latinos, early smoking was a predictor of high school dropout while alcohol and cannabis use were not[10,20]. Our findings indicated similar associations between school non-attendance and (i) current smoking without life-time cannabis or methamphetamine use, (ii) life-time cannabis use in addition to current smoking, (iii) and life-time methamphetamine use in addition to life-time cannabis use and current smoking.

The high degree of 'overlap' in substances used creates challenges for understanding the unique contributions of different substances to non-attendance at school. For example, 89% of life-time methamphetamine users in our study were also life-time cannabis users and 72% of life-time methamphetamine users were also current smokers, leaving too few students who used methamphetamine but had not used cannabis or did not smoke currently for analysis comparisons. More in depth research may be required to determine if methamphetamine use alone contributes significantly to high school dropout or absenteeism, given its documented detrimental cognitive and behavioural effects. Future studies may benefit from collecting more data on quantity and frequency of methamphetamine use and comparing non-attendance of frequent and regular users of methamphetamine with those who have only used it occasionally.

The limitations of our study include, firstly, that it is possible that some students may have under-reported substance use, despite our efforts to ensure confidentiality and anonymity. Secondly, while it is plausible that methamphetamine use leads to poor school attendance and/or early school drop-out, it is also possible that students with a lower academic aptitude, or other risk factors for poor academic performance (e.g. deviance and problem behaviours), are more inclined to use drugs, and this could account for the association between drug use and not attending school. Other than a question on repeating a year at school, our study did not measure academic performance or other behaviour problems shown to be related to dropout[21]. It is also possible that some of the students had moved and were hence enrolled in another school and still attending school. Our study was not able to capture this. Lastly, the present study was only able to examine life-time use of methamphetamine as there were too few students in the groups who had used methamphetamine more recently/frequently to conduct advanced analysis.

In conclusion, this study is the first we are aware of to investigate the impact of methamphetamine specifically on high school non-attendance. While an association between methamphetamine use and non-attendance was found and students who had used methamphetamine in addition to having used cannabis and smoking cigarettes currently had the highest odds ratio for non-attendance, we did not find this ratio to be significantly higher than those for students who reported current smoking but had not used cannabis or methamphetamine, and students who reported current smoking and had tried cannabis but not methamphetamine. Our study did support a serious consideration of the role of methamphetamine and other drugs in high-school non-attendance for policy makers. Early identification of students with methamphetamine and other substance use problems, and a supportive rather than punitive school policy, may be valuable in improving high school completion and student retention rates. In order to facilitate the identification of students with methamphetamine and other substance use problems and early intervention, a shift in government policy regarding the appointment and ratios of school psychologists and psychological services for students should perhaps be considered. These services and posts have been cut back in recent years, a policy which should perhaps be reconsidered.

## Competing interests

The authors declare that they have no competing interests.

## Authors' contributions

All authors have materially participated in the research and/or manuscript preparation. All authors have read and approved the final manuscript.

AP was the principal investigator of the study and primarily responsible for the drafting of the manuscript. AF, CP and RM were involved in the design of the study, revisions of the manuscript, and supervision of the study throughout. CL and RM assisted with statistical analysis and guidance on methods of analysis.
